# Practical Remarks on Gout, Rheumatic Fever, and Chronic Rheumatism of the Joints; Being the Substance of the Croonian Lectures for the Present Year, Delivered at the College of Physicians

**Published:** 1843-10

**Authors:** 


### Art. XIV.

*Practical Remarks on Gout, Rheumatic Fever, and Chronic Rheumatism of the Joints; being the substance of the Croonian Lectures for the present year, delivered at the College of Physicians.* By Robert Bentley Todd, m.d. f.r.s., Physician to the King's College Hospital, and Professor of Physiology in King's College, London.—*London,* 1843. Post 8vo, pp. 216.

We did not expect, when we recently addressed ourselves to the subject of gout and the disorders connected with it, to be so soon called upon to return to the consideration of the numerous and interesting questions it involves; but, desirous as we are to give our humble aid in forwarding any attempt to place pathology upon its true basis, and believing, as we do, that no disease can be so advantageously studied with this object as the one to which we now again invite the attention of our readers, we shall offer no apology for recurring to it. Indeed, the respectability of the authority from which the present treatise emanates, would of itself give to it strong claims on our attention, and on that of our readers. The author's purpose in laying his Croonian lectures before the public shall be stated in his own words.

"The object which the author has had in view, is to place in juxta-position the leading facts in the Natural History of Gout and Rheumatism, in order to direct the attention of practitioners, in a more especial manner than has hitherto been done, to what he conceives to be their true pathology. The work does not profess to give a complete history of these diseases; on the contrary, many details of symptoms, etiology, and treatment have been purposely omitted, as irrelevant to the argument, the design of which is, by contrasting the phenomena of gout and rheumatism with those of diseases confessedly caused by a morbid state of the fluids, to claim for them a similar origin. The author has availed himself of this opportunity of announcing some facts which he does not recollect to have been noticed by any previous writer on the subject. These will be found in the section "On the paroxysm of gout appearing in low states of the system;" and in that "On the rheumatic diathesis," where it is shown that disease of the heart may come on in that state of constitution, irrespectively of the occurrence of the rheumatic paroxysm, or fever." (Preface, pp. v-vi.)

Now we feel called upon to state, *in limine*, that we think the author has considerably over-rated the amount of novelty which his treatise contains. We should not have thought it necessary, at this time of day, to employ any arguments to establish a *claim* on the part of gout to a humoral origin; since we have always imagined that this is a subject long placed beyond dispute. Gout has been for a long period the strong hold of the humoral pathologist, even in the most exclusive reign of solidism; and from the time that the tide has begun to turn, and the morbific agency of a depraved state of the blood has been more attended to, gout has been continually cited as an unquestioned and apposite example of the influence of such a condition.* Moreover, the chemist has succeeded in detecting that which many pathologists have no hesitation in regarding (either in its simple state, or in combination with soda,) as the real *materies morbi*, lithic acid; and from the time that a strong probability (to say the least) was established in favour of this view by Mr. Murray Forbes, at the close of the last century, the treatment of intelligent practitioners has been directed to the expulsion of it from the system, and the prevention of its reaccumulation.

Dr. Todd attempts to unsettle our notions on this subject, however, by adducing facts and arguments which lead him to the inference that lithic acid is *not* the *materies morbi* of gout; but he does not substitute anything else in its place, his idea of the pathology of this disease being summed up in the following vague and indefinite proposition:

"It appears to me that we must look for the matter of gout as a compound, derived from a product of unhealthy action of the stomach and duodenum, which, being absorbed into the blood, unites there with some elements of the bile, which has been suffered to accumulate through the defective secretory action of the liver." (p. 74.)

As we shall presently join issue with Dr. Todd on this subject, we shall briefly recapitulate the principles which we advanced in our review of Dr. Bence Jones's Treatise, in regard to the causes of an accumulation of lithic acid in the blood.

I. The most frequent cause of an undue production of lithic acid in the system, we believe to be the ingestion of an excess of azotized food. If more is absorbed than can be applied to the replacement of the waste of the body, it must necessarily undergo decomposition, whilst still circulating in the blood; hence, if not drawn off by the excreting organs, it must accumulate in the vessels.

II. Even where there is no excess of production of lithic acid in the system, there may be an accumulation of it in the blood, in consequence of deficient excretion. The reduction of the acid to an insoluble form, by the removal of the

* See, for example, Dr. W. Budd's paper on the Symmetry of Disease, in the Medico-Chirurgical Transactions, vol. xxv.

ammonia with which it is usually combined, through the agency of another acid, is one of the most frequent causes of this deficiency. The presence of an undue amount of lactic acid in the blood, therefore, may operate as effectually in inducing the accumulation of lithic acid in the system, as the excessive production of the latter. The lactic acid may either be generated in undue amount, by disorder of the digestive process, or it may accumulate in consequence of deficient respiration and cutaneous excretion.

III. We recognize as a possible cause, though we cannot admit that any proof of its agency has yet been given, the introduction of an insufficient amount of oxygen into the system; so that the products of the decomposition of the muscular and other azotized tissues are partly converted into lithic acid, instead of into urea.

Now in regard to the constitutional treatment which we should found upon these principles, we pointed out the necessity, in the *first* place, of reducing the azotized portion of the diet to as low an amount as is consistent with affording the supply required to repair the waste of the body; but, *secondly*, we noticed the importance of strict attention to the due digestibility of the diet employed; since, if lactic acid be unduly generated in the stomach, we shall in vain attempt to get rid of lithic acid from the blood; and *thirdly*, we urged attention to those means by which the excreting processes are rendered more active; exercise, diaphoretics, purgatives, &c.,—the first of these serving the additional purpose of drawing off any surplus azotized matter in the blood, and at the same time of introducing an increased supply of oxygen. To these pathological and therapeutic principles, we shall more than once have occasion to refer in our subsequent remarks.

Dr. Todd commences with some judicious observations on the importance of studying the natural history of disease, and on the mode of advancing our knowledge of it. In regard to the particular maladies which form the subject of this treatise, he remarks:

"Upon neither the nature, nor the proper mode of treatment of either of these maladies, but more especially of the latter, can it be said that the views of medical men coincide. We can scarcely ascribe this disagreement of opinion to absolute ignorance of the natural history of these diseases, for they have long been the subjects of close and careful observation; and the descriptions of the older physicians have not been surpassed by the *longioris ævi diligentia*. The true cause of the difference seems to consist in an *imperfect analysis* of their natural history; so that we still stand in need of a careful induction from the facts with which the observations of practical physicians have supplied us, in connexion with the improved state of our knowledge of the phenomena of nutrition in its normal state." (p. 11.)

He then proceeds to advance the claim of gout and rheumatism to be regarded as diseases of the blood, in a manner which, as we have already remarked, would lead a previously ignorant reader to suppose that some considerable novelty was contained in the proposition, and that it was requisite for the author to "guard himself against the imputation of having stated merely speculative notions." Before describing the characters of the true *blood-diseases,* he makes some distinctions which it is important to keep in view.

"Let me, in order to prevent misunderstanding, separate from blood-diseases those which, originating in some local taint of nutrition, are disseminated by the blood to other and even distant parts of the body. The whole class of malignant diseases may be referred to this category; cancer, fungoid disease, &c., originate in a local derangement of nutrition, as in the stomach, the lip, the penis, &c., where the peculiar cancer-cells are first formed. If the local malady be within reach of the surgeon's knife, and can be removed early, the best chance is afforded to the patient of being rescued from its reappearance in some other place; but if, as is too often the case, the diseased part has been allowed to remain too long, the peculiar cancerous matter makes its way into the blood, and is carried along in the current of the circulation to other organs, where they become the nuclei of new cancerous formations." (p. 13.)

This distinction had been previously drawn by Dr. W. Budd, in his paper on cancer, (Lancet, 1841-2, vol. ii. p. 266;) and in terms so very similar, as evidently we think to have suggested the preceding paragraph. Dr. Todd then proceeds:

"We must also separate those diseases which appear to arise from the deficiency of some element which forms a necessary constituent of the blood, such as purpura, scurvy, and certain states of the body characterized by an anemic condition."

We are not quite sure how far purpura and scurvy can be characterized as diseases of mere *deficiency* of certain elements of the blood; but supposing them to be so, why does not Dr. Todd arrange under the same category diseases which result from an *excess* of certain normal constituents of the blood? Is it not equally certain that in the inflammatory diathesis there is an excessive production of fibrin, and that in the plethoric condition there is an excess of red corpuscles?

"In what I would call the *true blood-diseases*," continues Dr. Todd, "a morbid matter is generated by an abnormal chemical action in the blood itself. The morbid element may be formed primarily in the blood, in consequence of some check to one or more of the ordinary excretions, or from the supply of nutrient material being too great for the rate at which excretion is carried on; or it may have been introduced into the blood as a poison, which speedily deranges the normal changes that are continually going on within it. In this last way, all contagious diseases are propagated, the matter of contagion having been introduced into the blood; malarious diseases are also referrible to this cause. To either or both of the former causes, I hope to be able to show that rheumatism and gout are attributable." (p. 14.)

This is a proposition, we again repeat, which scarcely seems to us to require so laboured a demonstration, more especially after the elaborate and ingenious paper by Dr. W. Budd on Symmetrical Diseases (Medico-Chirurgical Transactions, vol. xxv.), to which in this, and several other parts of his treatise, Dr. Todd seems to us to lie under a degree of obligation, which is but insufficiently acknowledged by a reference to it in regard to the particular subject of the symmetry of chronic rheumatic affections (p. 133). For example, there is continual allusion throughout the work to the proneness of the morbific matters circulating in the blood to fix themselves upon particular parts to the exclusion of others, by a sort of affinity or attraction for the components of those parts. As a vague general hypothesis, this idea might be said to have been long floating in the minds of humoral pathologists; but it was brought forward by Dr. Budd (and by Mr. Paget) in a new and precise form, and supported by novel and very striking evidence; and the identical form, and much of the evidence, is employed by Dr. Todd without reference to the source from which he derived it. We are sure that Dr. Todd is far too honorable a man to have done this from any other cause than inadvertence; but we feel it to be a part of our duty as critics, to keep before the public mind the real sources of improved views which may arise from time to time, either as to the science or art of medicine; and where an author brings forward views which he has derived from others, in a form that *seems* (even though he may not so intend it) to claim for them his own paternity, we feel it to be an imperative act of justice to direct attention to their real sources. The following quotations, from among many similar parallelisms we could adduce, will serve, we think, as a justification of the tone we have adopted.

"From these facts it appears highly probable that there is a peculiar matter circulating in the blood, which give rise to the phenomena of gout, by occasioning a disturbance of longer or shorter duration, of greater or less intensity, in the nutrition of parts to which it is attracted; the effects of this morbific matter upon any part seem proportionate to the quantity that is drawn to the part; and when strongly attracted to one part, it is not likely to affect others. Hence in first attacks of gout in strong and healthy individuals, a single joint only is generally affected; but if anything impair the force of attraction of the morbid matter to that joint, the poison flies to other parts, which it will fix upon simultaneously or in succession.

"A safe and most important rule of practice may be derived from this fact; namely, *to be very cautious about interfering with the local disturbance which a fit of the gout creates in external parts.* For such local interference, by diminishing the force of attraction of the part first affected, for the gouty matter, may favour its transference to other places." (pp. 55, 56.)

"In the use of purgatives, care should be taken to employ only mild ones. Drastic purgatives should never be given; as, by the excessive irritation of the intestinal canal, which they would create, the gouty matter might be attracted to it from the external parts." (p. 102.)

"These views derive interesting confirmation from a fact to which many a gouty patient can bear testimony, namely, that a part which had previously been injured, is certain to be the seat of the gouty inflammation in subsequent attacks. If a foot or hand have been sprained or otherwise injured, the gout will fix itself there, because the injury has exalted the nutritive process of the part, and there is therefore a greater attraction of the elements of the blood to that part, than in the state of health.* For the essence of nutrition consists in an attraction between the solids and fluids, by which those elements of the latter which correspond in chemical constitution to the former, are drawn into and appropriated by them." (pp. 56, 57.)

We shall now subjoin a few extracts from Dr. Budd's paper; in which, it is to be remembered, the subject of gout as a well known blood-disease is only incidentally noticed, the author chiefly directing attention to the causes why it should not uniformly affect the body in that *symmetrical* manner which he shows to be characteristic of most blood-diseases, and to be one of their most important indications.

"It may be stated, then, as a general proposition, that, in diseases consisting of a number of lesions having a tendency to a symmetrical arrangement, the symmetry will be more perfect, as the course of the affection has been more free from febrile movement or local vascular excitement; in short, has been more chronic in progress, and has resembled more nearly in character the ordinary processes of assimilation; constituting, in this latter relation, a fact of striking import; and giving, in the view which regards these processes as consisting in the separation of matters of definite and identical composition from the blood, by structures of identical nature, the best authority for the particular theory of these diseases, maintained in the foregoing pages.

"Another circumstance of great effect in interfering with the manifestation of

* Cruveilhier states that those joints are most liable to be affected by gout which are most used.

symmetry in disease, consists in the influence which mechanical injury, or any other cause materially affecting the organic condition of a given part, has, in determining morbid matters present in the blood to that part, in preference to others of the same structure. The powerful effect of this influence is sufficiently familiar to practitioners. In gout, especially, this influence of mechanical injury is of such frequent and obvious effect, that M. Cruveilhier has been led to regard the friction and mechanical shocks to which the joints of the feet are especially subject, as the sole cause of the preference of gout for these parts." (Op. cit. p. 127.)

And again, subsequently, Dr. Budd remarks:

"From these views we may also learn the great importance in the treatment of diseases which are liable to shift their seat, of keeping the vital organs as far as possible in a quiescent state, lest, by exposing them to causes of irritation, we thereby favour the metastasis of the morbid matter to the irritated organ, and endanger the life of the patient. The neglect of this important precaution may be, I have several times seen reason to believe, the cause of the transference of gouty matter to the stomach, and the occasion of a fatal event." (p. 131.)

This therapeutic precept evidently corresponds, both in its purpose and its rationale, with that on which Dr. Todd lays so much stress, though couched in different terms.

We shall not again refer to this topic; and gladly quit it, to consider, with Dr. Todd, the origin of gout, and its connexion with the lithic-acid diathesis. As we have already remarked, he does not agree with Dr. Prout and other eminent authorities of the present time, in regarding lithic acid as the *materies morbi*, but his reasons for dissenting from them appear to us far from satisfactory. He seems entirely to lose sight of the fact, that gout results from an accumulation of lithic acid in the blood, whilst a deposit of lithic acid *in the urine* takes place in consequence of its separation from the blood. Hence, as we have already pointed out, we may have an accumulation of lithic acid in the blood, giving rise to gout, without any over-production, in consequence of deficient elimination; and, on the other hand, we may have a copious gravelly deposit of lithic acid in the urine, without the least tendency to accumulation of this substance in the blood. Hence gout and gravel may be vicarious, and it is well known to practitioners that they are frequently so. The symptoms that appeared to forewarn an attack of gout are often observed to pass away, when a copious discharge of lithic acid takes place by the urine, and a regular paroxysm of gout seems frequently to be relieved by a similar critical evacuation. Doubtless, gout and lithic-acid gravel may coexist; for, in consequence of an over-production of lithic acid, there may be more of it in the blood than the kidneys can possibly separate; and we may therefore have gouty symptoms developing themselves in the body, whilst there is at the same time a copious deposit in the urine. But where the deposit takes place, not from excess, but from precipitation, there is no reason whatever why gout should be expected; and the objection urged by Dr. Todd to the idea, that lithic acid is the *materies morbi* of gout—namely, that a copious deposit of this acid in the urine often takes place for weeks or months together, without any gouty symptoms manifesting themselves (p. 66)—is thus totally destitute of force. We are surprised that this view of the case did not occur to him, since he is well aware that an over-production of *lactic* acid in the stomach is one of the commonest causes of an attack of gout (p. 73); and the influence of this agent, in reducing the lithic acid to its insoluble form by depriving it of its base, ammonia, and thereby rendering it more difficult of excretion, has been long since pointed out by Dr. Prout, and has been generally recognized.

We find little else to remark upon in Dr. Todd's views of gout and its treatment, except the claim which he sets up (p. 42) to being the first to point out "that a low or depressed state of the system is favorable to the development of the gouty paroxysm." We believe the fact to be generally familiar to practitioners, and to have been noticed by writers on gout from the earliest times. Dr. Copland, in his learned Dictionary, when reciting the *predisposing* causes of the disease, includes the depressing passions and venereal excesses as "weakening nervous agency, and the functions of digestion and excretion;" and he assigns to these still greater force as *exciting* causes. "Cold seems to operate, partly by suppressing the excretions, and partly by depressing nervous power." All powerful mental emotions, whether exciting or depressing, will excite a paroxysm; but anger or vexation has this effect in a very remarkable degree. The ancients made anger to be the midwife of gout; and Cadogan considered vexation, in its wide signification, as one of his "three great causes of the disease." Besides these, Dr. Copland enumerates excessive bodily or mental labour; and in general, "whatever disorders the digesting and excretory organs, or suddenly impresses the nervous system." We quite agree with Dr. Copland in the *modus operandi* of these depressing causes; the disorder of the digestive apparatus which they induce having a tendency to generate lactic acid; and that of the deficient action of the excretory organs preventing the evacuation of the insoluble lithic acid thus set free, so as to occasion its accumulation in the blood. Hence the inference which Dr. Todd subsequently draws, (p. 73,) from the appearance of gout in low states of the system,—"that the morbid element of the disease may be present, independently of lithic acid," since, in the cases adduced by him, "this substance either did not abound, or did not exceed the normal quantity by a greater amount than may at any time be caused by a slight general disturbance,"—is entirely baseless. Dr. Todd has not proved that there was no accumulation of lithic acid *in the blood;* and there is a strong presumption that there was;—for in one of the cases mentioned, the individual was affected with Bright's disease of the kidney, and in others there was evident dyspepsia, resulting from an injudicious regimen.

Under the head of treatment we find little to remark upon. In regard to diet, Dr. Todd expresses views very similar to those advanced in our former article;—namely, that the azotized portion should be reduced as low as possible, consistently with the due maintenance of the bodily vigour; and that those kinds of food should be selected which are least liable to produce disorder of the digestive process. He lays considerable stress on the importance of promoting the secretions from the skin; on which point we fully agree with him; and he speaks highly, also, of the effects of copious diluents, not taken at the time of meals, but in the intervals. The following are his observations on the employment of colchicum; from which it will be seen that it differs entirely from those who attribute the greatest power of this remedy to its purgative effects. For ourselves we may say that, generally speaking, we agree with him; but that we have seen cases in which the most obvious benefit was derived from the more violent effects of the remedy, when its milder influence seemed to have given no decided relief.

"It appears to me that colchicum may act in a twofold manner: first, *chemically*, by producing some change in the urinary and hepatic secretions, both of which it tends to increase in quantity and alter in quality; and, secondly, it acts upon the nervous system, causing more or less depression, and on the mucous membrane of the stomach and bowels, exciting nausea, or vomiting, or purging, either separately or together. If employed in such doses as will produce only its chemical changes, it will, in strong constitutions, most favorably modify the gouty paroxysm, and certainly shorten its duration. If on the other hand, it produce any of its irritant effects, it is likely to do more harm than good; and therefore the dose should be diminished, or the medicine abandoned, if nausea or purging should come on during its administration. I have no doubt that a large share of the bad repute of this medicine is to be attributed to the indiscriminate and careless manner in which it is often prescribed; and I would venture to suggest the following hints for the guidance of the practitioner in its employment.

"1. Colchicum should not be given in the asthenic form of gout. [?]

"2. Colchicum should never be given at the outset of a paroxysm, nor until the bowels have been duly acted on by mild purgatives.

"3. The first doses of the medicine should be very small; they may be gradually increased.

"4. Colchicum should be always administered at first uncombined with any other medicine, until the practitioner has satisfied himself that it is not likely to disagree with his patient. And, indeed, there is always a disadvantage in administering this medicine in combination with others; since it may become difficult, if not impossible, at times, to determine what effects should be ascribed to the colchicum and what to the other ingredients.

"5. It should not be administered so as to excite nausea, vomiting, or purging. These effects should be regarded as indicative of the unfavorable operation of the medicine.

"6. Colchicum may be regarded as acting favorably when, under its use, the urine is increased in quantity, a more abundant bile is discharged; when the fæces, though solid, are surrounded by mucus, and the skin secretes freely.

"7. The effects of colchicum should be carefully watched; as, like digitalis and other medicines, it is apt to accumulate in the system.

"The use of this medicine seems chiefly applicable to the sthenic form of gout, which occurs in robust constitutions, and in the prime of life; but it is almost inadmissible in persons advanced in years, who have had several attacks, and in whom the malady would seem too deeply rooted to be influenced by the temporary administration of this remedy." (pp. 104-6.)

Before quitting the subject of gout, there is a suggestion which we think it desirable to throw out as to the nature of its *materies morbi.* We have hitherto spoken of it as *lithic acid;* but it occurs to us as more probable (though we would not venture to propound the idea dogmatically) that *lithate of soda* is the real morbific agent. This appears to us to be indicated by the fact, that the substance named is that which is separated from the blood in gouty deposits; and still more by the known connexion of gout with biliary as well as with urinary derangements, and by the beneficial result of treatment directed to *both* these excretions. Under the influence of particular substances, as we have seen, *lithic acid* has a tendency to accumulate in the blood; and it seems to us quite possible that, so long as it retains its uncombined form, gout may not result. But if, by deficiency in the secretion of bile, *soda* also be allowed to accumulate, the two will combine and lithate of soda will be formed. We offer this hypothesis for the consideration of our readers.

It accords well with the fact that peculiar advantage, in the prophylactic treatment of gout, is derived from the occasional administration of blue-pill or other mild mercurials.

Dr. Todd next proceeds to consider the natural history of rheumatism; and there are several points in his view of it which strike us as important. To these, therefore, we shall direct the special attention of our readers. He prefers the use of the term rheumatic fever to acute rheumatism; because the amount of febrile disturbance is frequently out of all proportion to the articular affection, and indicates the independent action of some morbid element in the blood; whilst in an ordinary local inflammation the febrile disturbance is constantly proportioned to its extent and intensity. This, we feel assured, is a correct and valuable distinction. He then directs attention to the importance of the fact that a *rheumatic diathesis* may exist without presenting the usual phenomena of rheumatism; and that, in this condition, the heart may become seriously affected, without the constitutional state having previously attracted the notice of the patient or his friends.

"I have now so frequently met with instances of diseased heart in young persons not traceable to an actual paroxysm of rheumatic fever, who nevertheless showed evident marks of a rheumatic diathesis which had existed for a longer or shorter time, that I cannot but regard this state of constitution as a fertile source of those cardiac diseases which are met with in early life. (p. 111.) . . . . . . . . I have not met with any allusion to the occurrence of disease of the heart, under these circumstances, in any of the works that I have had an opportunity of consulting. It is evidently a point of great value in the clinical history of the rheumatic diathesis, not only from the obvious bearing it has upon a correct view of the pathology of the disease, but also in a practical point of view, as showing how important it is for the practitioner to be on the watch for the signs of this state of constitution; as no doubt, if it could be removed, many young people might be saved from the terrible consequences of organic lesion of the heart. (p. 115) . . . . . . The occurrence of cardiac affection, as a feature of the rheumatic state of the constitution, must surely be admitted to be completely opposed to and utterly inexplicable by the doctrine of metastasis, which supposes that the cardiac inflammation has been transferred from the limbs to the heart. The truth is that the *cardiac inflammation may be primary;* it frequently exists at the same time with the articular affection, and dates its origin from the same period, as it derives it from the same cause." (p. 117.)

In this last statement, Dr. Todd is supported by the authority of Dr. Graves; and by the experience, we doubt not, of numerous practitioners. After urging other considerations in favour of the constitutional origin of rheumatic disorders, as being clearly traceable to a diseased state of the nutritive processes, (a position which few, we think, would now question,) Dr. Todd inquires whether the affection of the joints is really to be considered as *inflammatory* in its character.

"We have no satisfactory evidence that the articular affections are truly of the nature of ordinary inflammation. The parts are certainly swollen, painful, and there is a considerable flow of blood to them; but they do not suffer even the effusion of coagulable lymph; much less are they the subject of those destructive and disorganizing processes which so often follow in the wake of a true inflammation. Nor is it the nature of a true inflammation to desert quickly one part, leaving it unimpaired, and to fasten upon another; and, after making a short sojourn there, to revisit its old abode, or to fly to some new region. It is true that, in some instances, the joints do not escape unharmed; but a slow derangement of their nutrition is induced,—which may go on for years and years,—which alters the textures and even allows them to wear away, and which resists those remedies that usually check an inflammatory process. This, although often called inflammation of a chronic kind, is surely more like the slow and insidious working of a canker, which dries up or impairs the matter destined for the nourishment of the tissues.

"I do not wish to be understood as denying that the ordinary effects of inflammation may take place in rheumatic joints. I am bound to admit that some cases are on record which seem to partake of this nature, but such instances are extremely rare, and their occurrence does not invalidate my argument; on the contrary, here *exceptio probat regulam.* In these cases the poison has been more strongly attracted to a particular joint or joints, and others have suffered less; and the remarkable disposition which the rheumatic affection has to shift from joint to joint is absent." (pp. 135-6.)

Dr. Todd admits, of course, that true inflammation exists in the case of the heart; but he thinks that this may be induced by the constant motion of the organ, whilst in the state of irritation induced by the rheumatic affection; and that, if the joints could be freely used during the paroxysm, they would more frequently exhibit marks of destructive inflammation as a consequence of the rheumatic paroxysm. Our views of this subject must depend in part upon the import which we attach to the term *inflammation.* If we abide by the ancient definition, we shall find all its symptoms, heat, pain, redness, and swelling, present in the joint affected with acute rheumatism. But if we adopt the modern view, that it is a process essentially characterized by increase in the quantity of fibrin in the blood circulating through the part, and by the tendency to the effusion of this in the form of coagulable lymph, we may find it difficult to show, by examination of the joint, that such a change takes place in articular rheumatism, however acute its form. But the state of the blood seems to us unequivocally to indicate the existence of true inflammation *somewhere.* We are surprised that, on such an important topic, Dr. Todd should not have referred to the elaborate inquiries of M. Andral, instead of contenting himself with the following vague statement: "It is generally stated that the proportion of fibrin in the blood is much increased. As to the proportion of the red corpuscles, I should say that it must be considerably diminished." (p. 122.) He compares the state of the blood in rheumatism to that of anemic patients; thereby leaving it to be inferred, that the buffy coat is to be attributed to the same cause as that which produces it in *them;* namely, a diminished proportion of red corpuscles, rather than an increase in the fibrin. But what are the results of M. Andral's inquiries? Why that in acute rheumatism (a malady which, he remarks, differs greatly from ordinary inflammations, and yet agrees with them in this important particular,) the proportion of fibrin increases from 2½ or 3, to 4, 5, 6, 7, 8, 9, or even 10 parts in 1000; in the sub-acute form of the disease, it oscillates between 4 and 5; whilst in decidedly chronic rheumatism, it returns to its natural standard. Now we can quite imagine it as *probable* (though we do not see that it is *proved*) that the real *inflammation* is but secondary to the process in which the rheumatic affection essentially consists; just as inflammation may be secondary to tubercular deposit. But we think it an unwarrantable assumption that no inflammation exists in acute articular rheumatism. Moreover, in rheumatic inflammation of the eye—a disease of whose existence Dr. Todd expresses a doubt (p. 110), but which, from our own experience, we should say is just as evidently connected with the rheumatic diathesis as is affection of the heart, the deposition of coagulable lymph can be seen, and its subsequent absorption watched.

Dr. Todd then goes on to argue that the form of rheumatism termed *capsular* by Dr. Macleod is really to be considered as a modification of gout; and then proceeds to inquire into the nature of the morbid matter, to the presence of which in the blood the rheumatic diathesis is to be attributed. After the objections (invalid as we regard them) which he has adduced against the doctrine, that lithic acid is the *materies morbi* of gout, we are surprised to find him adopting, with little reserve, the idea of Dr. Prout that lactic acid stands in this relation to rheumatism. Without denying that it may be so, we shall only remark that the symptoms are by no means fully explained upon the supposition; and that, in particular, the absence of relief by the copious acid perspirations, which so often spontaneously break out during the attack, seems to indicate that the elimination of lactic acid from the blood does not draw off from it the morbid matter to which the affection is really due. Yet we would not deny the possibility of the accumulation of lactic acid in the blood being so great that not even these copious acid perspirations materially diminish it; and such an idea derives force from the beneficial result of the treatment of the hot-air bath, when so employed as to produce a vastly increased secretion from the skin. To this point we shall presently recur.

An interesting chapter then follows, on the connexion of rheumatism with uterine derangement, in which a considerable amount of evidence is adduced, both from direct observation and from analogy, to prove that the accumulation of rheumatic matter in the blood may be a not unfrequent result of defective uterine action. Drs. Locock and Rigby are cited as having observed rheumatic affections to be not unfrequent accompaniments of certain forms of dysmenorrhea. Additional evidence to this effect is brought forward in the succeeding chapter on chronic rheumatism of the joints, in which that peculiar alteration of the osseous texture and synovial membrane, which has been so well described by Mr. Adams of Dublin, is particularly adverted to. Some doubt exists as to whether this disease can strictly be said to be rheumatic in its character, for it is scarcely questionable that falls upon the great trochanter have given rise to the first symptoms of the disease, as was the case, we believe, with Charles Matthews, whose hip-joint we remember to have seen not long after his death. But, as Dr. Todd justly remarks, "this is by no means improbable; nor is the fact opposed to that view of the disease which assigns to it a rheumatic origin, for doubtless the perversion of nutrition, excited by the violence of the fall, would, as often happens in gout, occasion a greater attraction of the rheumatic matter to the injured joints, than would otherwise have taken place." In these cases, too, that symmetry is for the most part absent, which, as Dr. W. Budd has shown, is more remarkable in the purely constitutional forms of this disease, than in almost every other; the affection often repeating itself in the corresponding joints of the two sides with the most perfect exactness, and the distortion which it produces being identical.

Under the head of Treatment, we have little remark to make upon Dr. Todd's views. Consistently with his idea of the pathology of rheumatism, he trusts more to evacuants, which act by increasing the natural excretions, than to any specific remedies, or to depleting measures; and we believe that, although in particular cases, large bleedings may be indicated by the plethoric state of the system, (which, we may remark, is more frequently the case in the provinces than in the metropolis,) and although some practitioners have dwelt much upon the benefits derived from large doses of opium, Dover's powder, camphor, colchicum, calomel, and opium, &c., the most generally successful plan is that which Dr. Todd recommends, namely, a moderate bleeding at the commencement, serving to unload the vessels and to promote the action of medicines, and a combination of sudorifics, purgatives, and diuretics, especially such as are most efficacious in drawing off fluid from the system; diluents being at the same time freely allowed, in proportion to the patient's desire for them. We are much inclined to believe, from evidence which has recently come before us, that too much attention cannot be given to promoting the action of the skin in both the acute and chronic forms of this disease; and here the results of practice and those of theory coincide to a remarkable extent. For we know that the skin is the principal channel for the elimination of lactic acid from the system, and this especially in the rheumatic diathesis; if, therefore, lactic acid be the element which it is most desirable to remove from the blood, a further increase in the action of the skin would seem to be the obvious mode of effecting this. In so far, therefore, as the hydropathic system of treatment answers this end, we believe that it will be found an efficacious method of treating rheumatism. Several cases have come to our knowledge, in which great benefit has been derived from it, in the most obstinate chronic forms of the disorder; and the modification of it, which is employed by Dr. Freeman at Cheltenham, seems to us more desirable than the original plan of Preissnitz. The copious perspiration is induced, not by wrapping the patient in a wet sheet for five or six hours, but by placing him in a stove, heated up to 160° or 180°. There he remains, drinking as much fluid as he desires, until a copious perspiration (leaving behind three or four pints of fluid in the blanket that closely envelopes the body, and the oilskin on which the patient lies) breaks out; after which the patient takes the cold plunge, *à la Russe*, which we understand to produce the most delightful sensations. Such a plan would seem admirably calculated to draw off lactic acid, or any similar offending substance, from the blood; and of its utility we have had convincing testimony, particularly as regards the chronic forms of the disease, in which the structure of the joints is undergoing alteration. It has the beneficial effect of not only drawing off the *materies morbi*, but of preventing its re-formation, for the plan seems to operate most beneficially upon the digestive system, improving the appetite, strengthening the powers of the stomach, and correcting the secretions.

In closing our notice of Dr. Todd's work, we should be wrong if we were not to add that, although we have felt bound to express our difference from him on several points, and especially in regard to the degree of novelty which some of his views may claim, we can safely recommend his treatise to the student and junior practitioner, as containing much that they will not meet with elsewhere, and as suggesting to them many topics of inquiry, by following out which they may themselves do something to advance the knowledge of these most important diseases. We fully agree with Dr. Todd in the greater part of his general views. No one can be more alive than we desire to be to everything which can tend to the advance of medical science; and we believe that the reconstruction of the humoral pathology, upon the basis that will be afforded by organic chemistry, and by an improved mode of analysing disease, will be the most certain means of accomplishing this important end.

---

